# Dietary Garlic Powder Alleviates Lipopolysaccharide-Induced Inflammatory Response and Oxidative Stress through Regulating the Immunity and Intestinal Barrier Function in Broilers

**DOI:** 10.3390/ani12172281

**Published:** 2022-09-02

**Authors:** Ruiqiang Zhang, Jinsong Liu, Yulan Liu, Yanping Wu, Yinglei Xu, Jie Feng

**Affiliations:** 1 College of Animal Science, Key Laboratory of Animal Feed and Nutrition of Zhejiang Province, Zhejiang University, Hangzhou 310058, China; 2 College of Animal Science and Technology, College of Veterinary Medicine, Zhejiang Agricultural and Forestry University, Hangzhou 311300, China; 3 Key Agricultural Research Institute of Green Animal Health Products of Zhejiang Province, Zhejiang Vegamax Biotechnology Co., Ltd., Huzhou 313300, China

**Keywords:** garlic powder, lipopolysaccharide, inflammatory response, intestinal barrier function, broiler

## Abstract

**Simple Summary:**

This research was performed to determine the positive effects of GP on growth and intestinal function in lipopolysaccharide (LPS) challenged broilers. Results show that LPS challenge enhanced the weight loss rate, decreased the immunity and antioxidant capability, increased the intestinal permeability in broilers. When compared with LPS group, broilers fed with GP exhibited improved weight loss rate and jejunum villus height, enhanced ileum antioxidant function, and ameliorated intestinal barrier function. The LPS-challenged broilers in GP group had higher immunity than that of broilers in antibiotics group. In conclusion, GP supplementation could act as a natural alternative to antibiotic additive to alleviate the LPS-induced weight loss rate, inflammatory responses, and oxidative stress in broilers by improving the immunity and intestinal function.

**Abstract:**

Garlic powder (GP) has the outstanding antibacterial, antifungal, antiviral, anti-parasitic and antioxidant characteristics because of its various contained bioactive components, such as alliin, allicin, and polysaccharide, etc. It has been widely used as a native medicine and shown to prevent a variety of diseases. This research was performed to determine the positive effects of GP on growth and intestinal function in lipopolysaccharide (LPS) challenged broilers. A total of 480 one-day-old male Ross 308 broilers of similar initial body weight were randomly divided into four groups with 8 replicates per treatment and 15 chicks each replicate. LPS challenge enhanced the weight loss rate, decreased the immunity and antioxidant capability, increased the intestinal permeability in broilers. When compared with LPS group, broilers fed with GP exhibited improved weight loss rate and jejunum villus height, enhanced ileum antioxidant function, and ameliorated intestinal barrier function. The LPS-challenged broilers in GP group had higher immunity than that of broilers in antibiotics group. GP supplementation could act as a natural alternative to antibiotic additive to alleviate the LPS-induced weight loss rate, inflammatory responses, and oxidative stress in broilers by improving the immunity and intestinal function.

## 1. Introduction

Antibiotics were used in livestock industry as feed additives to enhance growth performance and preclude infectious diseases [1]. Although the excellent benefits in animals production is recognized well, the usage of dietary antimicrobial agents has caused potential adverse consequences, such as environmental pollution, drug residues in animal products, and the production of antibiotic resistance in pathogens [2]. In this regard, European Commission and China, have enacted the relevant laws to prohibit the use of antibiotics as growth promoters in animal diets [3,4]. Consequently, there is an urgency to explore available alternative feed additives to guarantee animal performance and health status.

Garlic, *Allium sativum*, has various bioactive components, such as alliin, diallylsulfides, and allicin, which show antibacterial, antifungal, antiviral, anti-parasitic and antioxidant characteristics [5]. Clinical studies have demonstrated that administration of garlic, as a native medicine, has therapeutic potential in diseases treatment [6]. Garlic-derived polysaccharide could alleviate colitis by improving mucosal barriers, blocking proinflammatory cytokines in mouse model [7]. Researchers found that feeding garlic powder (GP) to animals could improve productivity and meat quality, modulate the antioxidant and immune system, enhance health status, as well as decrease mortality rate [8,9,10]. Moreover, dietary GP has been confirmed to be salutary for growth performance, immunity, and intestine histology in birds, and the suitable level of GP inclusion in broiler diet is 1000 mg/kg feed [11,12]. Intestine tract is the most primary barrier in resisting pathogenic bacteria, viruses, and other harmful substances from entering the host. Intact and healthy intestine plays an essential role in maintaining animal health and production [13]. Regarding broiler, with the high stocking density and withdrawal of dietary antibiotics, the intestinal barrier is most sensitive to different stressors, specially immunological challenge [14]. However, for as we know, limited research was conducted to investigate the effect of garlic powder supplementation as an alternative to antibiotics on immune stress and intestinal health in broilers.

The lipopolysaccharide (LPS), a major molecule of the outer membrane in Gram-negative bacteria, is an endotoxin used in the well-documented model for immunological stress in animals [15]. Abdominally or orally injected LPS can trigger oxidative stress response, leading to the accumulation of reactive oxygen species, which then induces inflammation response, reconstructs of nutrient supply, impedes the growth rate, causes multiple diseases, and ultimately lead to death in mice [16], pig [17], and Arbor Acres broiler [18]. The mechanism of inflammatory response induced by LPS has been extensively explored. LPS conducts signal transduction through the transmembrane Toll-like receptor 4 (TLR 4), then activates the downstream cascades, such as nuclear factor kappa B (NF-κB), thereby promoting the biosynthesis of pro-inflammatory cytokines [19]. Thus, we used an LPS intraperitoneal injection to establish an immune injury model. This study was performed to evaluate the protective effects of dietary garlic powder inclusion on immunity and intestinal barrier function in response to the LPS challenge of broilers, and highlight the application of garlic powder as substitution to antibiotics in animals.

## 2. Materials and Methods

### 2.1. Animal and Treatment

The experiment procedures in the current research were approved by Zhejiang University Animal Care and Use Committee (NO. 21792). A total of 480 one-day-old male Ross 308 broilers of similar initial body weight (39.06 ± 0.22 g) were randomly assorted into 4 groups with 8 replicates per treatment and 15 chicks each replicate. The four treatments were as follows: (1) non-challenged broilers given a basal diet (CON group); (2) LPS-challenged broilers given the basal diet (LPS group); (3) LPS-challenged broilers fed with 75 mg/kg chlortetracycline supplemented diet (ANT group); and (4) LPS-challenged broilers fed with 1000 mg/kg GP supplemented diet (GP group). The basal diets were prepared and formulated to meet per nutrient requirement of NRC (1994) for broilers (Table S1). The chlortetracycline and GP were kindly offered by Zhejiang Vegamax Biotechnology Co., Ltd; Huzhou, China). During the feed preparation process, garlic powder and chlortetracycline were premixed with the commercial premix, respectively, which were then mixed with the formula feed in the form of mash. The experimental period was 44 d. At 42, 43, and 44 days of age, the birds injected intraperitoneally with LPS at a dose of 1 mg/kg body weight or equivalent volume of sterile saline solution at 8:00 a.m., respectively. The LPS from *Escherichia coli* was purchased from Sigma Chemical Co. (L2880, St. Louis, MO, USA). The dosage of LPS and its administration protocol performed in present study were referred to previous reported study [20].

### 2.2. Sample Collection

Three hours after the last LPS administration, one broiler from each replicate was selected at random for sampling. Blood sample was obtained by wing veinpuncture into vacutainers. The samples were collected, centrifuged, and stored at −80 °C. Subsequently, broilers were euthanized via cervical dislocation. The 1.5-cm sections of mid-jejunum and ileum were separated and fixed in 10% pre-cooled formalin for further histological analysis. The sterile glass microscope slide was used to sample the mucosal sample. The mucosa samples were then frozen using the liquid nitrogen and stored at −80 °C.

### 2.3. Body Weight

The broiler chickens were individually weighed before and after LPS challenge. The weight loss rate (WLR) was calculated as the following equation:

$$W\mathrm{LR}\;\left(\%\right)=100\times \frac{\;\mathrm{body}\;\mathrm{weight}\;\mathrm{before}\;\mathrm{LPS}\;\mathrm{challenged}\;-\;\mathrm{body}\;\mathrm{weight}\;\mathrm{after}\;\mathrm{LPS}\;\mathrm{challenged}}{\mathrm{body}\;\mathrm{weight}\;\mathrm{before}\;\mathrm{LPS}\;\mathrm{challenged}}$$

### 2.4. Serum Physiological and Biochemical Status

Serum levels of IgA, IgY, IgM, IL-4, IL-6, IL-10, IL-1β, and TNF-α were measured by enzyme-linked immunosorbent assay (ELISA) using corresponding reagent kit (Nanjing Angle Gene Bioengineering Co., Ltd., Nanjing, China) according to the manufacturer’s instructions. The diamine oxidase (DAO) and D-lactate acid (D-LA) in serum as biomarkers of intestinal permeability were measured by commercially available kits (Nanjing Angle Gene Bioengineering Co., Ltd., Nanjing, China) following the manufacturer’s instructions.

### 2.5. Intestinal Morphology Analysis

The jejunum and ileum segments were dehydrated in a graded series of ethanol, cleared with xylol, and embedded in paraffin. Paraffin blocks were sliced into 5 µm-thick sections using a rotary microtome. Then, every two sections for each segment were attached to glass slides and stained with hematoxylin-eosin. Intestinal morphology was observed employing a Nikon inverted microscope (Tokyo, Japan) equipped with a digital imaging analysis system. Six integrated villi and their associated crypts from each segment were measured for the villus height and crypt dept.

### 2.6. Intestinal Antioxidant Capacity

Mucosa sample from jejunum and ileum were homogenized with chilled 154 mmol/L sterile saline (1:4, wt/vol). The mixture was centrifuged at 4000× *g* for 10 min at 4 °C to acquire supernatant. Malondialdehyde (MDA), total superoxide dismutase (T-SOD), glutathione peroxidase (GPx), and catalase (CAT) levels in supernatants were determined to evaluate intestinal antioxidant status. The total protein concentration of each sample was also measured to normalize the aforementioned results. Specific diagnostic reagent kits were purchased from Nanjing Jiancheng Bioengineering Institute (Nanjing, China), and analyses were implemented according to the instructions of manufacturer.

### 2.7. Real-Time PCR Analysis

Total RNA was isolated from mucosal sample with RNAiso reagent (Takara Bio, Inc., Beijing, China) in strict accordance with the instructions. The level and quality of extracted RNA were determined using a Nano-300 microspectrophotometer (Hangzhou Allsheng Co., Ltd., Hangzhou, China). Then, reverse transcription of qualified RNA was carried out with PrimeScript™ RT Master Mix reagent kit on a gDNA Eraser (Takara Bio, Inc., Beijing, China). The primer sequences of target genes (*TLR2* = *Toll-like receptors 2*, *TLR4*, *MyD88* = *myeloid differentiation factor 88*, *NF-κB*, *IL 4* = *interleukin 4*, *IL-1β* = *interleukin-1β*, *TNF-α* = *tumor necrosis factor-α*, *MUC-2* = *mucoprotein 2*, *Occludin*, *Claudin-1*, *ZO-1* = *zonula occludens-1*) and internal control gene (*β-actin*) used in this research are shown in Table S2 and synthesized by TSINGKE Biological Technology (Hangzhou, China). RT-PCR kit (RR420A, Takara Bio, Inc., Beijing, China) was applied to execute the PCR program with CFX96 Real-Time System (Bio-Rad, Singapore). The cycle time values of target genes were normalized by that of control gene, and then calculated by 2^−ΔΔCt^ method to achieve the relative mRNA expression level.

### 2.8. Statistical Analysis

Raw data in this research were collected and preliminarily processed using Excel 2019. Result analysis was performed using one-way ANOVA in the statistical software SPSS version 22.0, Chicago, IL, USA. Significant differences among treatments were then determined by Duncan’s multiple range test. Figures were generated by the Prism software version 8.0. Overall data were presented as mean and SEM. *p* < 0.05 was set as a hallmark of statistical significance level.

## 3. Results

### 3.1. Body Weight

As shown in Table 1, there was no significant difference in body weight in broilers among treatments before and after the LPS injection (*p* > 0.05). LPS challenge increased WLR value when compared with the control group (*p* < 0.05). Both antibiotic and GP treatments decreased the WLR value of birds subjected to LPS (*p* < 0.05).

### 3.2. Serum Immunoglobulins

**Figure 1:**
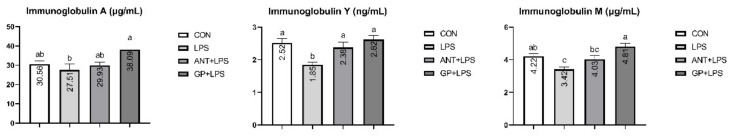
Effects of dietary garlic powder inclusion on serum immunoglobulins levels in broilers challenged with lipopolysaccharide (data were presented as mean and SEM, means that do not share the same superscript are significantly different, *p* < 0.05).

As indicated in Figure 1, broilers with LPS challenge exhibited lower serum IgY and IgM in than those in the control group (*p* < 0.05). Compared with the LPS group, a higher IgY content in serum was observed in antibiotic group (*p* < 0.05); dietary GP increased IgA, IgY, and IgM levels in serum of broilers (*p* < 0.05). In addition, GP supplementation enhanced the serum IgM content than that in antibiotic-treated broilers (*p* < 0.05).

### 3.3. Serum Inflammatory Cytokines

**Figure 2:**
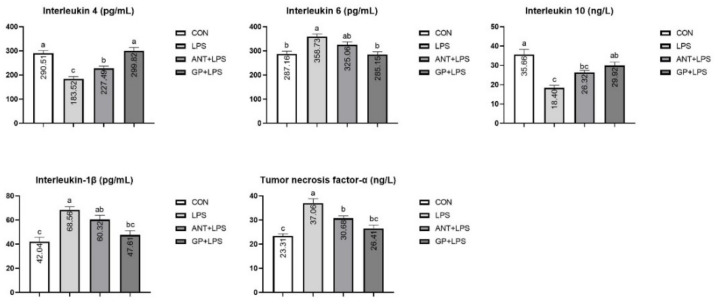
Effects of dietary garlic powder inclusion on serum inflammatory cytokines contents in broilers challenged with lipopolysaccharide (data were presented as mean and SEM, means that do not share the same superscript are significantly different, *p* < 0.05).

The LPS group showed lower anti-inflammatory cytokines (IL-4 and IL-10) contents in serum, whereas had higher serum pro-inflammatory cytokines (IL-6, IL-1β, and TNF-α) levels, which were reversed by dietary GP addition (*p* < 0.05, Figure 2). Moreover, dietary antibiotic addition had higher IL-4 content, and lower TNF-α content in serum of broilers in comparison with the LPS group, (*p* < 0.05). Moreover, the inclusion of GP in broiler increased the IL-4 content in serum when compared with the antibiotic treatment (*p* < 0.05).

### 3.4. Serum DAO and D-LA

**Figure 3:**
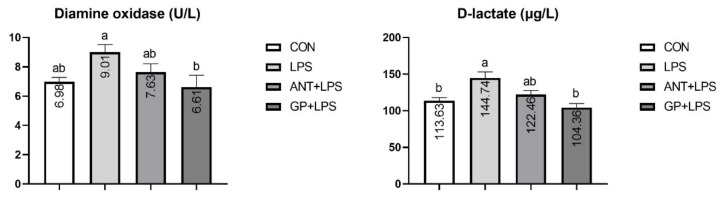
Effects of dietary garlic powder inclusion on serum diamine oxidase and D-lactate contents in broilers challenged with lipopolysaccharide (data were presented as mean and SEM, means that do not share the same superscript are significantly different, *p* < 0.05).

LPS injection increased the serum D-LA level in birds (*p* < 0.05, Figure 3). Broilers consuming GP inclusion showed reduced levels of D-LA and DAO in serum when compared with their LPS-challenged counterparts (*p* < 0.05). However, no effect was observed in serum D-LA or DAO by the antibiotic supplementation (*p* > 0.05).

### 3.5. Intestinal Morphology

**Figure 4:**
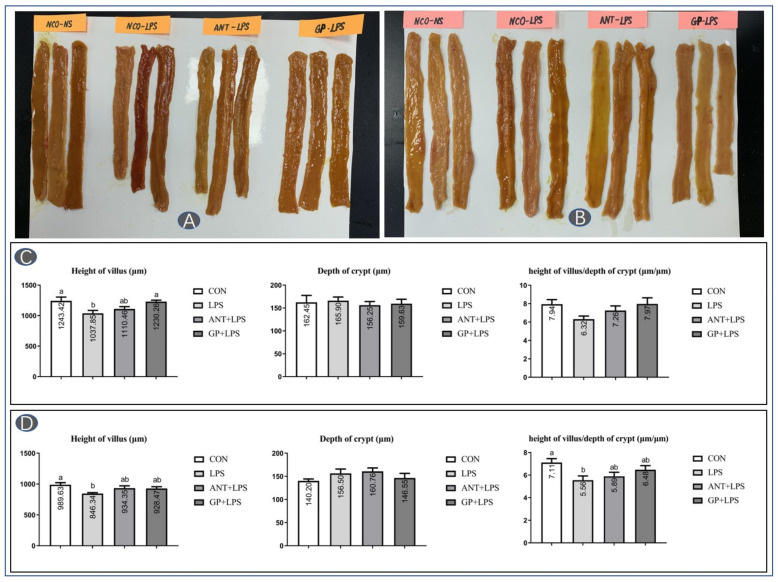
Effects of dietary garlic powder inclusion on intestine morphology ((**A**,**C**) jejunum; (**B**,**D**) ileum) in broilers challenged with lipopolysaccharide (data were presented as mean and SEM, means that do not share the same superscript are significantly different, *p* < 0.05).

The health status and morphology of intestinal mucosa in broilers are summarized in Figure 4. There were visual bleeding points in jejunum (Figure 4A) and ileum (Figure 4B) under LPS challenge. Supplemental antibiotic and GP attenuated the severity of intestinal histologic lesion in broilers. The jejunal villus height (Figure 4C), as well as ileal villus height and villus height/crypt depth value (Figure 4D) were decreased by LPS challenge (*p* < 0.05). Dietary GP alleviated the LPS induced reduction of junal villus height in broilers (*p* < 0.05).

### 3.6. Intestinal Antioxidant Status

**Figure 5:**
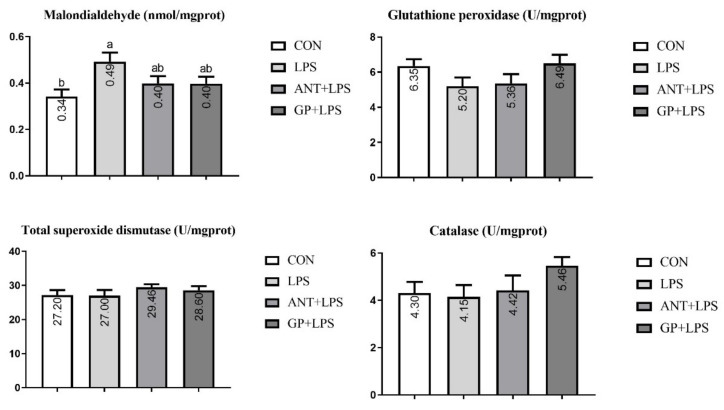
Effects of dietary garlic powder inclusion on antioxidant function of jejunum mucosa in broilers challenged with lipopolysaccharide (data were presented as mean and SEM, means that do not share the same superscript are significantly different, *p* < 0.05).

Results in Figure 5 showed that LPS injection increased the jejunal MDA accumulation in broilers (*p* < 0.05). There were no obvious effects on mucosal GPx, T-SOD, and CAT activities of jejunum in broilers among the four groups (*p* > 0.05).

**Figure 6:**
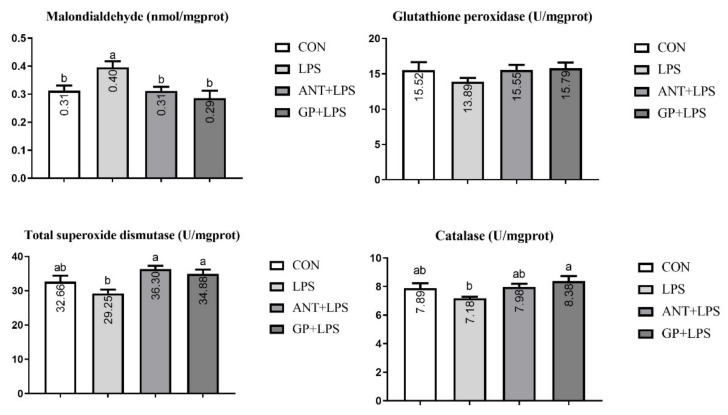
Effects of dietary garlic powder inclusion on antioxidant function of ileum mucosa in broilers challenged with lipopolysaccharide (data were presented as mean and SEM, means that do not share the same superscript are significantly different, *p* < 0.05).

As presented in Figure 6, ileal MDA was increased in the LPS challenge group as compared to the unchallenged group, and the higher content was counteracted by antibiotic and GP supplementation (*p* < 0.05). For antioxidant enzymes, SOD activity in antibiotic group, and levels of SOD and CAT in GP group were improved in the jejunum of LPS-challenged broiler, respectively (*p* < 0.05).

### 3.7. Intestinal Mucosa Gene Expression

**Figure 7:**
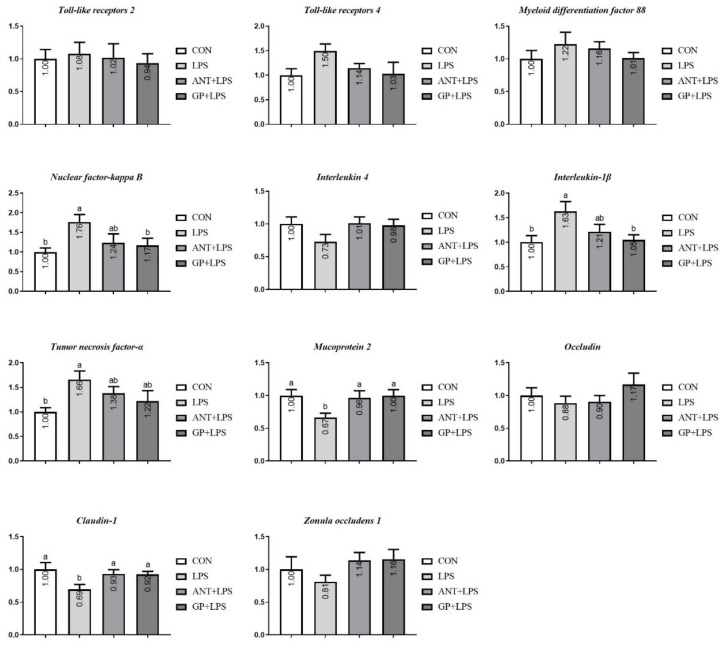
Effects of dietary garlic powder inclusion on relative gene expression of inflammatory cytokines and tight junction in jejunum mucosa of broilers challenged with lipopolysaccharide (data were presented as mean and SEM, means that do not share the same superscript are significantly different, *p* < 0.05).

For jejunal tissues, LPS challenge upregulated the mRNA abundance of *NF-κB*, *IL-1β*, *TNF-α*, and downregulated the mRNA expression levels of *MUC-2* and *Claudin-1* (*p* < 0.05, Figure 7). Compared with the LPS injected broilers, a suppression in the antibiotic group was observed for the mRNA abundance of *Claudin-1* and *MUC-2* (*p* < 0.05); broilers consuming GP-added diets had downregulated mRNA expressions of *NF-κB* and *IL-1β*, and upregulated mRNA expressions of *Claudin-1* and *MUC-2* (*p* < 0.05).

**Figure 8:**
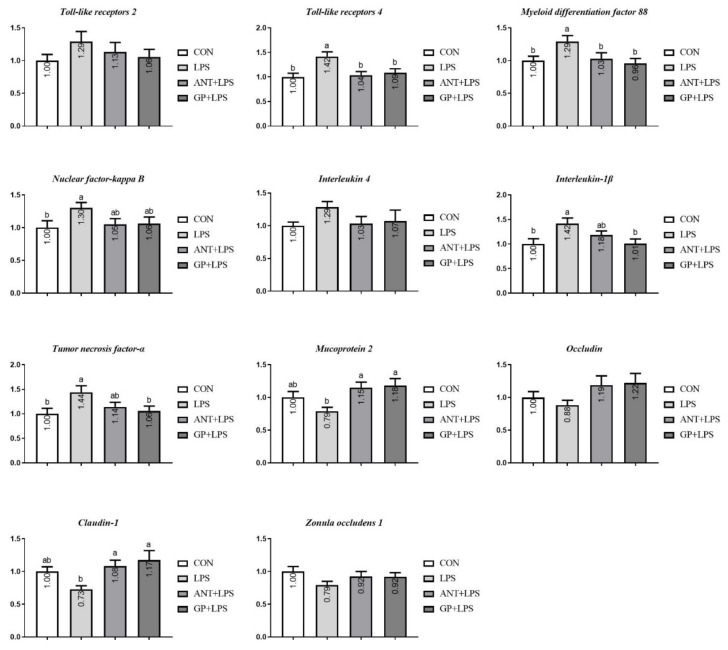
Effects of dietary garlic powder inclusion on relative gene expression of inflammatory cytokines and tight junction in ileum mucosa of broilers challenged with lipopolysaccharide (data were presented as mean and SEM, means that do not share the same superscript are significantly different, *p* < 0.05).

For ileal mucosa, broilers suffering LPS challenge exhibited reductions in *TLR4*, *MyD88*, *NF-κB*, *IL-1β*, and *TNF-α* mRNA expression levels than those in the control group (*p* < 0.05, Figure 8). Compared with the LPS individuals, antibiotic addition downregulated the abundance of *TLR4* and *MyD88* mRNA, upregulated expressions of *MUC-2* and *Claudin-1* mRNA (*p* < 0.05); GP supplementation upregulated the abundance of *MUC-2* and *Claudin-1*, and downregulated expressions of *TLR4* and *MyD88*, *IL-1β* and *TNF-α* mRNA (*p* < 0.05). In addition, the mRNA expressions of all determined genes in both jejunum and ileum achieved the similar level between the antibiotic and GP groups, respectively (*p* > 0.05).

## 4. Discussion

Numerous studies have confirmed that LPS challenge could result in poor compromised growth performance in broilers, as characterized by the depressed feed intake, body weight gain, and feed efficiency. It has been hypothesized that the immune stress induced by LPS injection could redistribute nutrients to synthesize antibody and inflammatory cytokine and elicit immune response, which ultimately lead to the growth retardation [18,21,22]. As expected, this study found that LPS injection increased the weight loss rate in broilers response to the established immune stress. The GP supplementation resulted in a decline of the weight loss rate in broilers challenged with LPS. Previous reports have demonstrated that dietary GP could promote the growth performance of broiler chickens [9,12,23]. Antibacterial compounds in garlic, such as allin, may contribute to the improvement in growth parameter when broilers are fed with GP [24]. Moreover, GP supplementation appeared to be as efficient as antibiotic in broiler growth, as evidenced by comparable body weight and weight loss rate. These results indicated that GP administration may represent a valid alternative to antibiotic to prevent the detrimental impacts on the growth performance in broilers under immunological stress.

The immune system is an importantly sophisticated defense mechanism to protect the body against exogenous material, and combat invasion by pathogenic agents [25]. Serum immunoglobulin is defined as a major part of immune system, and is involved in humoral immunity to maintain the immune balance [26]. Injection of LPS was reported to inhibit the production of immunoglobulin in B cells, which may attribute to the lower serum immunoglobulins in broilers with LPS treatment [27]. Feeding GP-included diet increased serum IgA, IgY, and IgM in broiler compared to LPS control group, implying that dietary GP could enhance broiler immunity to resist against LPS-induced immune dysfunction. The improvement in immunoglobulins may benefit from the bioactive molecules in garlic, such as allicin, allin, ajoene, and sallycysteine. For instance, administration of allicin was reported to alleviate reticuloendotheliosis virus-induced immunosuppression in broilers [28]. Moreover, with the presence of garlic supplementation, broiler had better immune function and immune organs development, as illustrated by improved serum immune parameters and increased immune organ weight [8,29]. Moreover, this study also found that antibiotic inclusion only enhanced the serum IgY levels in LPS-challenged broilers, indicating that GP was more advantageous than antibiotics on the protective effect in broilers challenge with LPS.

Generally, LPS can be recognized by the receptor protein in immune-related cells, and cause an acute inflammatory immune response in host [30]. TLR4 is a vital sensor for LPS, and can trigger the intracellular association of MyD88 and then activate NF-κB pathway, which subsequently lures the generation and release of inflammatory cytokines [31]. The signal pathway of TLR4/NF-κB is a complex process involved in the inflammatory response and characterized by a cascade of the expression of both pro-inflammatory (TNF-α, IL-6, and IL-1β) and anti-inflammatory (IL-4 and IL-10) cytokines [32]. In the model of LPS, we found IL-4 and IL-10 in serum were detected to be decreased, whereas contents of detected pro-inflammatory cytokines were raised, which are consistent with a previous study by Tong et al. [32]. Excessive inflammatory response has already been associated with growth impairment and immune disorder [33], and similar variations were presented in our study. Moreover, the imbalance productions of inflammatory cytokines were inverted by GP inclusion in broiler diet. Simultaneously, GP treatment exhibited the increased serum IL-4 level than antibiotic inclusion. Results obtained herein demonstrated that dietary GP is more effective than antibiotic for mitigating LPS-induced inflammation in broilers. Similarly, in a study based on cyclophosphamide-induced immunosuppression model, rats fed diet with garlic extract showed an improvement in anti-infection immunity, since the contents of pro-inflammatory cytokines (TNF-α and IL-1β) were declined and anti-inflammatory cytokines (IL-12 and INF-γ) levels were elevated in the serum [34]. LPS-induced intestinal inflammation could negatively impact gastrointestinal barrier structure and function in broilers [35]. In current experiment, LPS challenge boosted the expression of intestinal mucosa *TNF-α* and *IL-1β* with the concomitant upregulation of *TLR4*, *MyD88*, and *NF-κB* in broilers. Furthermore, the increased expression of *TLR4*, *MyD88*, *NF-κB*, *TNF-α*, and *IL-1β* triggered by LPS were inhibited by dietary GP. Therefore, dietary GP could suppress the LPS-induced excessive synthesis and production of pro-inflammatory cytokines in intestine. Antibiotic treatment, however, only exhibited inhibitory effects on the mRNA expression levels of *TLR 4* and *MyD88* in LPS injected broilers. Taken together, we speculated that dietary GP supplementation may be more pronounced than antibiotic additive to ameliorate the LPS-induced intestinal dysfunction via regulating the signal pathway of TLR4/MyD88/NF-κB in broilers.

It is well-known that the intestinal histomorphology is a reliable indicator for intestinal health status in animal [36]. In this trail, broilers suffering from LPS injection displayed apparent bleeding points in jejunum and ileum tissues, suggesting that LPS challenge triggered histological damage in intestine. The intestinal lesion severity was clearly relieved by dietary GP supplementation. Micromorphological characteristics of intestinal mucosa, including villus height, crypt depth, and villus height to crypt depth ratio, are commonly employed to assess nutrient absorptive property in broiler chicken [37]. In the LPS-challenged birds, the villus height was reduced in jejunum and ileum, and a decreased villus height/crypt depth value was found in ileum. Similar observation in intestinal morphology were reported in birds under immune stress [27]. Additional GP successfully reversed the compromised intestinal morphology in broilers challenged by LPS, because of the increased villus height, demonstrating that absorptive process and growth efficiency could be promoted in broilers receiving GP addition. Previous studies have proved that garlic powder, garlic meal, and garlic derivatives have exhibited a wide spectrum of antibacterial activity and then promote the intestinal health status, which may contribute to the improved intestinal morphology in GP-supplemented broilers [38,39]. Hence, we suggested that dietary GP supplementation, as a replacement to antibiotic, could be efficient to attenuate immune stress-induced intestinal injury and regulate intestinal morphology in birds.

The serum D-LA and DAO are certified as molecular marker for intestinal permeability [40]. When intestinal barrier is ruined and permeability increases, circulating D-LA and DAO levels increase in broiler chickens [41]. In our study, results confirmed that LPS treatment was associated with the higher DAO content, which indicated that the intestinal barrier in broilers was injured by the LPS injection. The reduction in serum D-LA and DAO levels in LPS-challenged broilers after GP supplementation inferred that GP could abate intestinal permeability, lessen the intestinal injury induced by LPS. In addition, there was only a tendency for antibiotic addition to reduce D-LA and DAO levels, implying that dietary GP in broilers could be a good alternative to antibiotic growth promoter to protect the integrity of intestinal mucosa from LPS challenge. Tight junctions, such as occludins and claudins have been identified as crucial transmembrane proteins in intestinal mucosa, sustaining paracellular permeability [42]. MUC2 is a main part of the intestinal mucus layer and plays an important role in avoiding intestinal mucosal injury [42]. We found that broilers exhibited up-regulations of jejunal *MUC-2* and *Claudin-1* response to LSP challenge, which was consistent with previous study [43]. Dietary GP inclusion enhanced the jejunum and ileum *MUC-2* and *Claudin-1* mRNA expression levels, which is in keeping with the D-LA and DAO declines of serum in broilers. Given the above, dietary GP may exert excellent influences to intestinal barrier defense.

Oxidative stress is considered a subject health problem in broilers which would cause protein modification, DNA damage, and immuno-pathological damage to animals [44]. Antioxidant system acts a vital role in resisting the undesirable effects induced by oxidative stress in animals [45]. MDA is the main metabolite of lipid peroxidation and used to assess the degree of oxidative stress injury [46]. GPx, SOD, and CAT are the most critical enzymes in antioxidant defense systems, which can protect the organism against detrimental pro-oxidants [47]. In the current study, the MDA accumulations in jejunum and ileum mucosa were enhanced in LPS-challenged broilers, indicating that oxidative stress in the intestine was induced by the LPS injection. Furthermore, dietary GP and antibiotic supplementation could alleviate the oxidative stress in broilers induced by LPS challenge, as evidenced by the decreased MDA level, increased SOD activity in comparison with the LPS-challenged control group. Increased CAT activity was observed in broilers with GP inclusion compared with the LPS group. Numerous compounds of GP such as allyl cysteine, alliin, allicin, and allyl disulfide are known to possess anti-oxidant activity against free radicals and prevent lipid peroxidation [48]. Previous studies also reported that garlic administration can improve the antioxidant status in rats [49], pigs [50], and broilers [8]. These findings indicated that dietary GP could attenuate the oxidative stress injury induced by LPS challenge, which may be due to the improvement in intestinal antioxidant function in broilers.

## 5. Conclusions

Dietary GP supplementation could attenuate the adverse impacts of LPS challenge on weight loss rate, inflammatory response injury, and oxidative stress in broilers, which might be mediated by improving serum immunoglobulins levels, regulating intestinal antioxidant status and intestinal mucosal barrier function. In addition, the supplementation of GP may be more beneficial than dietary antibiotic to protect against LPS-induced injury in broilers. Overall, this study suggests the potential of GP to replace antibiotic growth promoter and reduce immunological stress in broiler chicken.

## Figures and Tables

**Table 1:** Effects of dietary garlic powder inclusion on the body weight in broiler challenged with lipopolysaccharide.

Items ^†^	CON	LPS	ANT + LPS	GP + LPS	SEM	*p* Value
IBW, g	2402.29	2472.75	2587.08	2640.13	40.39	0.145
FBW, g	2227.50	2110.00	2282.50	2353.75	35.53	0.091
WLR, %	7.22 ^c^	14.60 ^a^	11.73 ^b^	10.88 ^b^	0.57	<0.001

Means do not share the same superscript in each column are significantly different (*p* < 0.05). ^†^ IBW, average body weight before challenged; FBW, average body weight after challenged; WLR, weight loss rate, CON, broilers fed a basal diet and challenged with sterile saline solution; LPS, broilers fed a basal diet and challenged lipopolysaccharide; ANT + LPS: broilers fed a basal diet supplemented with 75 mg/kg chlortetracycline and challenged with lipopolysaccharide; RLS + LPS: broilers fed a basal diet supplemented with 1000 mg/kg garlic powder and challenged with lipopolysaccharide.

## Data Availability

The data presented in this study are available on request from the corresponding author.

## Supplementary Materials

The following supporting information can be downloaded at: https://www.mdpi.com/article/10.3390/ani12172281/s1, Table S1: Composition and nutrient levels of basal diets (air-dry basis, %); Table S2: Primer sequences for real-time PCR assay.
