# Supplementary material for: Dietary Garlic Powder Alleviates Lipopolysaccharide-Induced Inflammatory Response and Oxidative Stress through Regulating the Immunity and Intestinal Barrier Function in Broilers
Source: Animals (Basel). 2022 Sep 2;12(17):2281. doi: 10.3390/ani12172281 (PMC9454656; doi:10.3390/ani12172281)
Supplement: Supplementary file 1 [file animals-12-02281-s001.zip › animals-1889401-supplementary.pdf]

Table S1. Composition and nutrient levels of basal diets (air-dry basis, %).

Items	Contents	
	1 to 21 days of age	22 to 42 days of age
Ingredients		
Corn	52.5	54
Soybean meal	25	17
Extruded soybean	4.5	3.5
DDGS	8.5	7.5
Rice bran		6
Corn gluten		2
Soybean oil	1.7	4.6
Limestone	1.4	1.4
Fermented soybean meal	2.4	
Premix [1]	4	4
Total	100.00	100.00
Nutrient levels		
CP	22.02	19.11
ME（MJ/kg）	12.23	12.91
EE	5.5	8.6
Lys	1.18	0.97
Met	0.54	0.45
Met+Cys	0.88	0.74
Thr	0.86	0.71
Try	0.23	0.20
Ca	0.82	0.73
TP	0.65	0.57

[1] The premix provided the following per kg of diets: V_A 10 000 IU, V_{B1} 2.2 mg, V_{B2} 8.0 mg, V_{B5} 40 mg, V_{B6} 4.0 mg, VB_{12} 0.013 mg, V_{D3} 3 000 IU, V_E 30 IU, V_{K3} 1.3 mg, biotin 0.04 mg, folic acid 40 mg, *D*-pantothenate calcium 10 mg, nicotinic acid 40 mg, choline chloride 400 mg, Cu 7.5 mg, Fe 80 mg, Mn 110 mg, Zn 65 mg, I 1.1 mg, Se 0.3 mg.

Table S2. Primer sequences for real-time PCR assay.

Gene[1]	Genbank id	Primer sequence, sense/antisense	Product size, bp
β-actin	NM_205518.1	TGCTGTGTTCCCATCTATCG<br>TTGGTGACAATACCGTGTTCA	150
TLR2	AB046533	CATTCACCATGAGGCAGGGATAG<br>GGTGCAGATCAAGGACACTAGGA	157
TLR4	NM_001030693.1	TGACCTACCCATCGGACACT<br>CTCAGGGCATCAAGGTCTCC	171
MyD88	NM_001030962.4	GATGATCCGTATGGGCATGGA<br>ATGGACCACACACACGTTCC	170
NF-κB	XM_015285418.2	TGCCTTTTGCTTGAGGGTGATG<br>CTGCCAGTTTTGTGAAGCCC	100
IL-4	NM_001007079.2	AGCACTGCCACAAGAACCT<br>GCTAGTTGGTGGAAGAAGGTAC	160
IL-1β	NM_204524.1	GTACCGAGTACAACCCCTGC<br>AGCAACGGGACGGTAATGAA	112
TNF-α	NM204267.1	CCGTAGTGCTGTTCTATGACCG<br>GTTCCACATCTTTCAGAGCATCAA	235
MUC-2	XM_001234581.3	AGGAATGGGCTGCAAGAGAC<br>GTGACATCAGGGCACACAGA	77
Occludin	NM_205128.1	AGCCCTCAATACCAGGATGTG<br>CGCTTGATGTGGAAGAGCTTG	125
Claudin-1	NM_001013611.2	CACACCCGTTAACACCAGATTT<br>GAGGGGGCATTTTTGGGGTA	159
ZO-1	XM_015278980.2	GGATGTTTATTTGGGCGGCT<br>CCATTGTTGCACTCTTGCCG	153

[1] *TLR2, 4=toll-like receptors 2, 4; MyD88=myeloid differentiation factor 88; NF-κB=nuclear factor-kappa B; IL-4= interleukin-4; IL-1β=interleukin-1β; TNF-α=tumor necrosis factor-α; MUC-2=mucoprotein 2; ZO-1=zonula occludens-1.*
